# Correlation of Mean Platelet Volume and Red Cell Distribution Width With HbA1c and Its Association With Microvascular Complications in Type 2 Diabetes Mellitus: A Cross-Sectional Study at a Tertiary Hospital in India

**DOI:** 10.7759/cureus.66139

**Published:** 2024-08-04

**Authors:** Ajay Dev, Nanda Kumar R, Arun K, Gisha Sivan, Raji Rajesh Lenin, JS Kumar

**Affiliations:** 1 General Medicine, SRM Medical College Hospital and Research Centre, SRM Institute of Science and Technology (SRMIST), Chengalpattu, IND; 2 General Medicine, SRM Medical College Hospital and Research Centre, Chengalpattu, IND; 3 Internal Medicine, SRM Medical College Hospital and Research Centre, Chengalpattu, IND; 4 Medical Research, SRM Medical College Hospital and Research Centre, SRM Institute of Science and Technology (SRMIST), Chengalpattu, IND

**Keywords:** retinopathy, complications, glycosylated hemoglobin, type 2 diabetes mellitus, red cell distribution width, mean platelet volume

## Abstract

Introduction: Diabetes mellitus type 2 (T2DM) is a metabolic disorder, and its prevalence is rising worldwide. The objective of the study was to investigate the association between mean platelet volume (MPV) and red cell distribution width (RDW) and the glycemic control marker HbA1c. So MPV and RDW could be used as prognostic indicators of deterioration of gluco-regulation in diabetes mellitus type 2 and the associated microvascular complications.

Methodology: A cross-sectional study was conducted on 216 type 2 diabetic patients, who were divided into two groups based on HbA1c values (<7% and >7%). Red blood cell distribution width, mean platelet volume, plasma glucose estimation, fasting lipid profile, spot urine albumin creatinine ratio (ACR), direct ophthalmoscopic examination, and nerve conduction study were tested in all the patients.

Results: Of the 216 individuals diagnosed with type 2 diabetes mellitus, 210 exhibited inadequate glycemic control, establishing a statistically significant correlation with triglyceride levels, mean platelet volume, and blood sugar levels. The study revealed a significant association between MPV and RDW and HbA1c levels. Additionally, microvascular complications such as retinopathy, proteinuria, and neuropathy exhibited strong correlations in this patient cohort, emphasizing the interconnectedness of glycemic control and various health indicators in individuals with T2DM.

Conclusion: This study provides significant results that mean platelet volume and red cell distribution can be used as markers in the diagnosis of microvascular complications in type 2 diabetes mellitus.

## Introduction

Type 2 diabetes, the most prevalent type of diabetes mellitus, is marked by chronic hyperglycemia, which can harm several organs and tissues, most notably the kidneys, eyes, peripheral nerves, and blood vessels [1]. It is extremely important for healthcare professionals to adopt a holistic approach that includes dietary and lifestyle modification, adequate exercise, appropriate medications, regular monitoring of blood glucose levels, timely follow-up, and a thorough assessment of complications. Microvascular and macrovascular complications of T2DM pose an increased risk to the patient's health. HbA1c, or glycated hemoglobin, has been used to assess blood sugar management in most cases. None of the commonly prescribed medications or any acute stressors that tend to affect blood glucose metabolism have an impact on HbA1c values [2]. There are additional benefits to fasting blood glucose readings as well, such as the fact that HbA1c has relatively low biological variability and is less affected by pre-analytical factors. Along with the glycosylation of hemoglobin, elevated blood glucose levels have several other negative effects on red blood cells that ultimately change their structure and hemodynamic properties [3]. These impacts include a rise in the adhesive properties, a decrease in the deformability, and a modification in the mechanical properties of RBCs. It was well understood that glycemic control is a much better approach to treating type 2 diabetes than glycemic management.

For a better understanding of the prognosis of diabetic patients, researchers have suggested using the mean platelet volume (MPV) and red cell distribution width (RDW) for assessing glycemic control [4]. Dense granules discovered in larger platelets exhibit elevated metabolic and enzymatic activity when compared to those observed in smaller ones, thus possessing a heightened potential for thrombosis. Consequently, an augmented mean platelet volume (MPV) and red cell distribution width (RDW) may also denote an increased propensity for thrombosis. Studies are conducted to establish a correlation between an elevated MPV, RDW, diminished platelet count, and endothelial dysfunction, including conditions such as metabolic syndrome, diabetes, coronary artery disease (CAD), and malignancies [5]. Disturbances in metabolic processes (such as increased blood sugar levels and elevated levels of triglycerides), as well as systemic dysfunctions (such as oxidative stress, inflammation, and impaired response to insulin), are some of the contributing factors in individuals with diabetes, particularly those with type 2 diabetes mellitus (T2DM), that result in heightened platelet reactivity. The implementation of appropriate glycemic control measures involving reducing platelet activity holds the potential to prevent or delay the occurrence of vascular complications in T2DM patients. Notably, the identification of altered red blood cell distribution width (RDW) levels contributes to the diagnosis and monitoring of glycemic status and its associated complications, given that elevated levels of glycated hemoglobin (HbA1c) have been established as a leading cause of the increased incidence of microvascular complications in diabetes mellitus [6]. So, the present study was undertaken to understand the correlation between MPV, RDW, and glycemic control in type 2 diabetic patients and also establish their association with microvascular complications, if any.

## Materials and methods

This is a prospective, cross-sectional study conducted on a sample of 216 type 2 diabetes mellitus patients who underwent treatment with insulin or oral hypoglycemic drugs at the tertiary hospital in Chennai, India.

Patients with a diabetic history were employed for the study. After obtaining their written consent, the patient’s history and demographic characteristics were recorded, and venous samples were taken for analysis of RBC distribution width, mean platelet volume, HbA1c, triglyceride levels, and serum creatinine levels after a fasting period of 12 hours. To understand microvascular complications, microalbuminuria, and ophthalmic examinations were conducted to study retinopathy, and monofilament tests, nerve conduction studies, ankle tendon reflexes, etc., were conducted to assess neuropathy. The eGFR CKD, using the EPI formula, was used to assess the patient's creatinine clearance.

The patients were categorized into two cohorts according to their HbA1c levels: individuals with commendable glycemic regulation and those lacking the same. A comparative examination of the mean platelet volume and red cell distribution width was subsequently performed between these two cohorts.

The data was analyzed using IBM Corp. Released 2015. IBM SPSS Statistics for Windows, Version 23.0. Armonk, NY: IBM Corp. For categorical variables, frequency analysis and percentage analysis were employed to summarize the data, while for continuous variables, means and standard deviations were calculated. To test for a statistically significant difference between the bivariate samples of the two groups, an unpaired t-test was carried out. The multivariate analysis was done using a one-way ANOVA. In order to determine whether or not a set of categorical data is statistically significant, the Chi-Square test was used. A p-value of less than 0.05 was considered significant.

## Results

Among the cohort of 216 patients studied, there was an almost equivalent distribution between the male demographic (50.7%) and the female demographic (49.3%). The mean age of the patients was 53.7 (Table 1). An examination conducted within the various glycemic control cohorts revealed that individuals who were 50 years old or younger showcased glycemic control in comparison to those who were over the age of 50. Out of the entire patient cohort, 210 individuals manifested inadequate glycemic control, while a mere 15 individuals displayed a favorable prognosis. When BMI was compared, better glycemic control was observed in patients with a BMI greater than 29.2 (obese individuals) than in the underweight population. In the subjects with fasting blood sugar levels greater than 140 mg/dl (n=214), around 96.2% exhibited poor glycemic control. The mean PPBS value in those with poor glycemic control was also higher (280+57.1) than in those with good control (234.7+53.2). The correlation of mean platelet volume with glycemic control showed a significant association with the mean MPV in the poor control group of 10.3 (SD=1.5) and the good control group of 9.2 (SD=1.1). The red cell distribution width values were also higher in the poor glycemic control subjects (13.4+1.4) than in others (12.7+1.4) (Table 2). Abdominal diameter has not shown any significant difference in both groups.

**Table 1:** Means and standard deviations of variables with the glycemic control BMI: Body mass index, FBS: Fasting blood sugar, PPBS: Post prandial blood sugar, MPV: Mean platelet volume, RDW: Red cell distribution width

	GLYCEMIC CONTROL		
Parameters	Good	Poor	p-value
	Mean +SD	Mean	
Age (years)	52 + 8.3	55.4 + 9.7	0.187
BMI (kg/m2)	29.2 + 4.2	28.6 + 4.4	0.608
FBS (mg/dL)	175.3 + 38.2	206.7 + 47	0.012*
PPBS (mg/dL)	234.7 + 53.2	280 + 57.1	0.003*
Triglycerides (mg/dL)	141.8 + 9.5	154.2 + 27.6	0.001*
MPV (fL)	9.2 + 1.1	10.3 + 1.5	0.007*
Abdominal diameter (cm)	96.9 + 5.2	96.3 + 6.9	0.755
RDW	12.7 +1.4	13.4 + 1.4	0.07

**Table 2:** Comparison of complications with the glycemic control

Variable	Glycemic Control		P value
	Good	Poor	
Diabetic Retinopathy			0.862
Present	9	130	
Absent	6	80	
Proteinuria			0.171
Present	3	85	
Absent	12	125	
Neuropathy			0.886
Present	8	116	
Absent	7	94	
Hypertension			0.710
Present	6	74	
Absent	9	145	

Hypertriglyceridemia was not seen in 86.7% of the subjects with a good prognosis, and it was present in only 38.1% of the poor control subjects. However, the mean value was higher in the poor control group. Similarly, hypertension was also seen in 35.2% of the poor control group, and this association was not significant.

The correlation of MPV score with HbA1c showed an R-value of 0.324 and a significant association with glycemic control (Table 3). The RDW and MPV values also showed great statistical significance, with a very strong positive correlation and an r-value of 0.491 (p<0.0005). Table 3 shows the correlation between MPV and RDW with diabetic retinopathy, neuropathy, nephropathy, and hypertension. Hypertension was not associated significantly with either MPV or RDW values in this study. Red cell distribution width did not seem to change in those with diabetic neuropathy (p>0.05) but is significantly associated with retinopathy and proteinuria (p<0.05). However, MPV was found to be significantly associated with all the complications, with a strong correlation. 

**Table 3:** Correlation of MPV and RDW with microvascular complications MPW: Mean platelet volume, RDW: Red cell distribution width

Variable	MPV	p-value	RDW	p-value
Diabetic Retinopathy		0.004		0.005
Present	10.47 (1.5)		13.5 (1.26)	
Absent	9.87 (1.46)		12.9 (1.6)	
Proteinuria		0.0005		0.037
Present	10.71 (1.53)		13.2(1.8)	
Absent	9.93(1.42)		12.4 (1.51)	
Neuropathy		0.034		0.075
Present	10.43 (1.46)		13.5 (1.40)	
Absent	10 (1.54)		13.1(1.44)	
Hypertension		0.176		0.328
Present	10.42 (1.55)		13.7(1.17)	
Absent	10.14 (1.49)		13.5(1.2)	

## Discussion

The majority of the patients with poor glycemic control fell between the ages of 52 and 60 in our study. A similar study found that as age increases, the prognosis of diabetes, especially type 2, will decrease. [7]. The same study linked the longer duration of having diabetes with concomitant hypertension and obesity, which, coupled with poor glycemic control, will result in a high risk of death and disability due to microvascular complications.

Poor glycemic control was seen in 41.4% of obese patients; similar findings identified that reducing body weight by 5% to 10% decreased HbA1c levels and other cardiovascular risk factors [8]. FBS and PPBS showed a significant correlation with the HbA1c levels in our study. A meta-analysis found that PPBS had a much higher connection with HbA1c than FBS, and a considerable decrease in postprandial glucose level was responsible for a higher decline in HbA1c levels than a reduction in fasting blood sugar levels [9].

Contrary to our studies on the association of hypertension with glycemic control, a study showed that lowering blood pressure levels decreased the incidence of vascular disease and mortality among diabetic patients, which was statistically significant [10]. Hypertriglyceridemia was seen in 38.1% of the study subjects with poor glycemic control, and it was also found that insulin-treated patients with diabetes mellitus were more likely to have high triglyceride levels. The results were similar to a study where they found that those with high triglyceride levels were more likely to have poor glycemic control. 40% of those with poor glycemic control had proteinuria in our study, and tight glycemic control significantly reduces the risk of microalbuminuria in people with type 2 diabetes mellitus [11]. This study found significance between diabetic retinopathy and glycemic control among the two groups. It was very well known that poorly controlled glucose levels lead to faster progression of the retinopathy. Similarly, neuropathy is seen in 55.2% of the patients with poor glycemic control, and this finding is consistent with a study that showed strict glycemic control is superior to glycemic management in preventing diabetic neuropathy [12].

An increase in mean platelet volume is associated with inadequate glucose management. It is expected that increased platelet activity owing to aberrant event action contributes to the development of vascular problems in type 2 diabetes mellitus [13]. A study demonstrates that people with diabetes have an abnormally large mean platelet volume, and the average volume of a person's platelets is larger in diabetics than in healthy individuals. MPV had a significant association with all the microvascular complications, as explored in a meta-analysis [11,14]. When RDW was compared with proteinuria and diabetic retinopathy, a strong positive connection was established in our study. The beta cell function measured by HOMA 2 was discovered to be correlated with RDW. A study compared RDW with micro- and macrovascular complications in diabetes and found that the chance of one diabetic complication increased up to 21% with RDW [15].

For a comprehensive understanding of MPV and RDW as reliable biomarkers for diabetes across various demographics and stages, a larger and more diverse cohort is essential. A multicentric study would further address variability in patient populations and clinical settings, offering a more comprehensive understanding of the role of MPV and RDW in the monitoring and management of diabetes and its cardiovascular complications.

## Conclusions

The risk factors for diabetic complications, such as longevity, glycemic control, and dyslipidemia, are all very well known. With an HbA1c value of 7 or higher, we observed a rise in MPV and RDW values in diabetic patients. This suggests that elevated MPV and RDW scores can be used as biomarkers to identify complications of diabetes before they arise, rather than HbA1c levels. However, more research is required to determine whether vascular problems are at the root of or a consequence of increased MPV. Therefore, we suggest that MPV be used as a straightforward and affordable instrument to track the development and management of DM and its cardiovascular complications.
